# Combined Use of RT-qPCR and NGS for Identification and Surveillance of SARS-CoV-2 Variants of Concern in Residual Clinical Laboratory Samples in Miami-Dade County, Florida

**DOI:** 10.3390/v15030593

**Published:** 2023-02-21

**Authors:** Yamina L. Carattini, Anthony Griswold, Sion Williams, Ranjini Valiathan, Yi Zhou, Bhavarth Shukla, Lilian M. Abbo, Katiuska Parra, Merce Jorda, Stephen D. Nimer, Corneliu Sologon, Hilma R. Gallegos, Roy E. Weiss, Tanira Ferreira, Abdul Memon, Peter G. Paige, Emmanuel Thomas, David M. Andrews

**Affiliations:** 1 Department of Pathology and Laboratory Medicine, University of Miami Miller School of Medicine, Miami, FL 33136, USA; 2 John P. Hussman Institute for Human Genomics, University of Miami Miller School of Medicine, Miami, FL 33136, USA; 3 Sylvester Comprehensive Cancer Center, University of Miami Miller School of Medicine, Miami, FL 33136, USA; 4 Department of Medicine, University of Miami Miller School of Medicine, Miami, FL 33136, USA; 5 Laboratory Services, Pathology Department, Jackson Memorial Hospital, Miami, FL 33136, USA; 6 Jackson Health System, Miami, FL 33136, USA

**Keywords:** SARS-CoV-2, COVID-19, delta, VOC, RT-qPCR, surveillance

## Abstract

Over the course of the COVID-19 pandemic, SARS-CoV-2 variants of concern (VOCs) with increased transmissibility and immune escape capabilities, such as Delta and Omicron, have triggered waves of new COVID-19 infections worldwide, and Omicron subvariants continue to represent a global health concern. Tracking the prevalence and dynamics of VOCs has clinical and epidemiological significance and is essential for modeling the progression and evolution of the COVID-19 pandemic. Next generation sequencing (NGS) is recognized as the gold standard for genomic characterization of SARS-CoV-2 variants, but it is labor and cost intensive and not amenable to rapid lineage identification. Here we describe a two-pronged approach for rapid, cost-effective surveillance of SARS-CoV-2 VOCs by combining reverse-transcriptase quantitative polymerase chain reaction (RT-qPCR) and periodic NGS with the ARTIC sequencing method. Variant surveillance by RT-qPCR included the commercially available TaqPath COVID-19 Combo Kit to track S-gene target failure (SGTF) associated with the spike protein deletion H69-V70, as well as two internally designed and validated RT-qPCR assays targeting two N-terminal-domain (NTD) spike gene deletions, NTD156-7 and NTD25-7. The NTD156-7 RT-qPCR assay facilitated tracking of the Delta variant, while the NTD25-7 RT-qPCR assay was used for tracking Omicron variants, including the BA.2, BA.4, and BA.5 lineages. In silico validation of the NTD156-7 and NTD25-7 primers and probes compared with publicly available SARS-CoV-2 genome databases showed low variability in regions corresponding to oligonucleotide binding sites. Similarly, in vitro validation with NGS-confirmed samples showed excellent correlation. RT-qPCR assays allow for near-real-time monitoring of circulating and emerging variants allowing for ongoing surveillance of variant dynamics in a local population. By performing periodic sequencing of variant surveillance by RT-qPCR methods, we were able to provide ongoing validation of the results obtained by RT-qPCR screening. Rapid SARS-CoV-2 variant identification and surveillance by this combined approach served to inform clinical decisions in a timely manner and permitted better utilization of sequencing resources.

## 1. Introduction

Throughout the COVID-19 pandemic, SARS-CoV-2 has continuously acquired mutations throughout its genome, resulting in the emergence of over a thousand genetic variations of the original Wuhan strain, categorized into lineages and larger phylogenetic groups, or clades [1]. A subset of these variants have been designated as variants of concern (VOC) by public health organizations including the WHO [2], the Centers for Disease Control and Prevention (CDC) [3], and the European Centre for Disease Prevention and Control (ECDC) [4] based on clinical and epidemiological data and on the presence of one or more mutations of interest, predominantly in the spike (S) glycoprotein (e.g., D614G, N501Y, N417K, E484K/Q, L452R, T478K, and P681R). Such mutations have been associated with increased transmissibility and/or virulence, enhanced ability to evade protective immunity from natural infection or vaccination, and/or reduced efficacy of therapeutic monoclonal antibodies [5,6,7,8,9]. Genetic mutations of interest may confer additional functional properties to the virus and have emerged through parallel evolutionary selection in several different lineages without defining a particular lineage or strain. In March 2022, the WHO and the CDC recognized two VOCs, Delta (B.1.617.2, including AY lineages) and Omicron (B.1.1.529, including BA. lineages) [3,10]

The Delta variant emerged in India in late 2020 and was classified as a VOC by the WHO in May 2021. With increased transmissibility and enhanced immune escape capabilities compared to previous variants [11,12,13,14], Delta caused waves of new COVID-19 infections around the world and rapidly outcompeted other lineages and VOCs including Alpha (B.1.1.7) and Gamma (P.1), achieving dominance in most countries by the fall of 2021 [15]. Soon after the culmination of the Delta-driven wave of COVID-19 infections, the Omicron variant emerged in South Africa with an unprecedented and alarming number of mutations in the S glycoprotein and was subsequently designated a VOC by the WHO on 26 November 2021 [16]. Of approximately 33 S gene mutations in present in the Omicron variant, 15 are in the receptor-binding domain (RBD), a region which represents the primary target of monoclonal antibody therapies and most SARS-CoV-2 vaccines [17]. More transmissible and better at immune evasion than previous variants [18,19,20,21], Omicron rapidly became the dominant strain throughout the world and drove the global number of new COVID-19 infections from 269 million in mid-December 2021 to more than 430 million by late-February 2022. The number of global COVID-19-related deaths rose from 5.3 million to over 6 million in the same period [22]. Iketani et al. showed disparate efficacy of monoclonal antibodies against different Omicron lineages (BA.1 vs. BA.2) [23], which rapidly changed the therapeutic landscape. Additionally, through its increased transmissibility and immune evasion properties, Omicron is characterized by high susceptibility to breakthrough infection, even in fully vaccinated individuals [24]. As Omicron continues its genetic evolution, ongoing surveillance of locally circulating lineages is necessary in order to monitor for the emergence of novel strains designated as Omicron subvariants under monitoring by the WHO. As of 13 September 2022, new COVID-19 cases in the United States (US) are predominantly of the Omicron BA.5 sublineage (~89%), with BA.4 comprising ~10%, and BA.2.12.2 representing <1% [25].

Although NGS is recognized as the gold standard for SARS-CoV-2 variant identification, it is cost- and labor-intensive, limiting its widespread use [26]. In addition, the turnaround time for results can take as much as 1 week to be finalized given the complex workflows. Furthermore, widely used multiplex-polymerase-chain-reaction (PCR)-based sequencing approaches (i.e., ARTIC SARS-CoV-2 sequencing method) harbor inherent technical limitations compared to more robust sequencing approaches. One limitation of the ARTIC method is high sequencing failure rates for samples with PCR Ct values >32 [27]. Another limitation associated with this method is primer-variant overlap with heavily mutated lineages (e.g., Omicron), which can result in PCR amplification failure and a subsequent decrease in coverage [28,29,30,31]. In contrast, PCR-based approaches represent a simpler approach to track specific VOCs [32]. Molecular techniques involving PCR have played an important role in COVID-19 diagnostic testing and are widely used for viral nucleic acid detection due to their high sensitivity. Importantly, PCR-based tests are also more amenable to providing semi-quantitative results pertaining to viral load [33].

In addition to their utility in COVID-19 diagnostic testing, PCR-based methods provide a feasible alternative to NGS for SARS-CoV-2 variant detection and surveillance. A commercially available Reverse Transcriptase quantitative PCR (RT-qPCR) COVID-19 diagnostic test (TaqPath COVID-19 Combo Kit, Thermo Fisher, Inc., Waltham, MA) became a useful tool for tracking the Alpha (B.1.1.7) variant in the first half of 2021, after discovery that a 6-base-pair (bp) deletion at nucleotide positions 21,765–21,770 in the N-terminal domain of the S gene, corresponding to amino acids (aa’s) Histidine-69 and Valine-70 (H69-V70) of the S glycoprotein, results in failed or delayed amplification (defined here by a mean S-gene target cycle threshold (Ct) delay = 6.3 compared to the N-gene target Ct), referred to as S-gene target failure (SGTF) or S-gene target late amplification (SGTL), while amplification of the other two targets in the assay (N-gene and ORF1ab-gene) are retained [34,35]. More recently, the prevalence of VOC Omicron lineages BA.1, BA.4, and BA.5, which also harbor the H69-V70 S deletion, have been successfully tracked by this method. We found excellent correlation between SGTF and SGTL with the TaqPath assay and NGS-confirmed Alpha and Omicron lineages (Ct <30), supporting the continued use of the TaqPath assay for tracking the prevalence of SARS-CoV-2 lineages carrying the H69-V70 deletion. In contrast to the Omicron BA.1, BA.4, and BA.5 lineages, the BA.2 lineages lack the H69-V70 S deletion that results in SGTF/SGTL and cannot be detected with the TaqPath assay. Other investigators have proposed RT-qPCR strategies targeting lineage-defining mutations as a reliable complement to NGS for detection and surveillance of SARS-CoV-2 variants [36,37,38,39,40]. Here we utilized a combined approach involving the TaqPath assay, targeted RT-qPCR, and NGS to follow changes in the SARS-CoV-2 variant pool. 

## 2. Materials and Methods

### 2.1. Study Design

Deidentified, residual SARS-CoV-2-positive nasal and/or nasopharyngeal swab (NPS) samples (in transport media/solution) were used in this study. SARS-CoV-2 samples previously identified as positive by PCR were retrieved randomly from routine COVID-19 PCR testing performed in local clinical laboratories. From January 2021 to April 2022, we obtained 6482 residual COVID-19-positve samples for SARS-CoV-2 variant screening by PCR. Of these, 1668 samples were submitted for genomic characterization by NGS. Residual patient samples were obtained from two health systems in Miami-Dade County, Florida: University of Miami Health System (UHealth) and Jackson Health System (JHS), a large public county health system distributed throughout Miami-Dade County. Residual samples were also obtained from routine University of Miami COVID-19 surveillance screening and testing programs involving students and staff. All samples used in this study were permanently de-identified and unlinked from the patient or student/staff records and were used for surveillance purposes only. Consequently, study samples were considered by the Institutional Review Board (IRB) to represent non-human studies research material and did not require IRB approval.

### 2.2. NGS/ARTIC SARS-CoV-2 Sequencing Method

Sample sequencing was performed at the University of Miami Miller School of Medicine, Sylvester Comprehensive Cancer Center Onco-genomics Shared Resource (OGSR) facility using the NEBNext ARTIC SARS-CoV-2 FS Library Prep Kit (Illumina, San Diego, CA, USA) (NEB #E7658), according to manufacturer’s instructions. Briefly, following reverse transcription of purified viral RNA, multiplexed PCR amplification of cDNA with a primer scheme produces multiple amplicons (~98) spanning the viral genome. Preparation of final PCR products for sequencing follows with barcoded adaptor ligation and addition of index primers in accordance with Illumina library preparation instructions. Sequencing was performed on the NextSeq 500 (Illumina, San Diego, CA, USA).

### 2.3. Bioinformatics

Bioinformatics analysis of raw FASTQ files was performed using the resources of the Institute for Data Science and Computing and performed by personnel at the John P. Hussman Institute for Human Genomics, both at the University of Miami Miller School of Medicine. The protocol used was based on a pipeline instituted at the Utah Department of Health and made publicly available via the Centers for Disease Control and Prevention GitHub page [41]. Secondary analysis of the resulting consensus FASTA file was performed in a two-part approach. First, the FASTA file was analyzed using Phylogenetic Assignment of Named Global Outbreak LINeages software (Pangolin https://github.com/cov-lineages/pangolin). Prior to each analysis, the version of Pangolin and its associated software and databases were updated to the most current version to account for software evolution and recently emerging annotations. The second part utilized the Nextclade web-based software (all versions: 10.5281/zenodo.5726680) [42]. Nextclade performs alignment of the FASTA file against the SARS-CoV-2 reference and quality control, calls variants, and assigns clades for each sample. A visual output of coverage and variant calls are provided as well as a tabular format for cataloging lineage information.

### 2.4. RT-qPCR Target Selection and Primer/Probe Design

To develop a targeted RT-qPCR assay that could distinguish Delta from other circulating variants, we searched SARS-CoV-2 Delta variant sequences retrieved from the GISAID database and subsequently selected a 6 bp deletion located in the NTD of the S gene as the target for the fluorescence resonance energy transfer (FRET) probe (qPCR 5-prime nuclease probe). The target mutation spans three codons and results in a 6 bp deletion of glutamate (E) at position 156 and phenylalanine (F) at position 157, also resulting in the non-synonymous substitution of arginine (R) for glycine (G) at position 158. We downloaded SARS-CoV-2 reference sequences from GISAID (hCoV-19/Wuhan/WIV04/2019) and NCBI (NC_045512.2) (https://www.gisaid.org/) to select the location and obtain the sequence for the primers and probe. We then modified the reference sequence for probe binding to the E156-F157 six-base-pair NTD deletion at nucleotide positions 22,029–22,034 and selected the location of the primers to ensure an amplicon size < 100 bp. Oligos were obtained from Integrated DNA Technologies (IDT, Carolville, IA) and were optimized in silico using the IDT OligoAnalyzer tool for determining melting temperatures (Tm) and potential for formation of secondary structures (hairpins or primer-dimer). The 24-base FRET probe was labeled at the 5′-end with the reporter molecule 6-carboxyfluorescein (FAM) and with a double quencher, ZEN (internal), and Iowa Black (3IABkFQ) at the 3′-end. The amplicon size for the NTD156-7 RT-qPCR assay is 96 bp. Using the same approach, we designed a mutation-specific RT-qPCR assay to detect and monitor the local prevalence of the Omicron BA.2, which subsequently served for surveillance of the BA.4 and BA.5 lineages as well. In this case, the NTD 9 bp deletion selected as the target spans four codons resulting in a non-synonymous substitution of leucine (L) with serine (S) at position 24, and deletion of two proline (P) and one alanine (A) residues at positions 25–27 of the S glycoprotein. The 22 base FRET probe was labeled at the 5′-end with the reporter molecule Cyanine 5 (Cy5) and with TAO and Iowa Black (3IAbRQSp) as internal and 3′-end quenchers, respectively. The amplicon size for the NTD25-7 assay is 88 bp. 

### 2.5. In Silico Validation of Targeted RT-qPCR Assays

To determine the rate of occurrence of mutations in the selected primers and probe sequences, we utilized the PrimerChecker function provided by the GISAID EpiCov resource (https://www.gisaid.org/). In short, the PrimerChecker performs a search using basic local alignment search tool (BLAST) parameters for short sequence matches to search input primer sequences against high quality (<1% N and <0.05% non-synonymous mutations) genome sequences deposited in GISAID. We analyzed sequences deposited from 23 February 2021 to 22 August 2021 (1,305,468 sequences) for the NTD156-7 primers and probe and from 19 December 2021 to 19 March 2022 (220,000 sequences) for the NTD25-7 primers and probe. These searches provided a list of sequences with one or more mutations in the binding regions for primers and FRET probes and captured the cumulative mutation rate during the period analyzed. 

### 2.6. In Vitro Validation of Targeted RT-qPCR Assays

To validate the primers and FRET probe for the Delta-specific RT-qPCR assay (NTD156-7) in vitro, we obtained extracted RNA from 50 COVID-19-positive samples of variants previously identified by NGS. RNA extractions were performed on a Chemagic 360 instrument using the Chemagic Viral DNA/RNA 300 Kit H96 (PerkinElmer, Downers Grove, ILfollowing manufacturer’s instructions. Of the 50 samples, *n* = 24 were identified as Delta variant (B.1.617.2 (9), AY.2 (1), AY.3 (3), AY.3.1 (2), AY.4 (1), AY.5 (1), AY.12 (1), AY.24 (1), AY.25 (5)), and *n* = 26 were non-Delta variants (B.1 (1), B.1.1.7 (Alpha) (6), B.1.427 (2), B.1.429 (2), B.1.526 (3), B.1.621 (Mu) (2), B.1.621.1 (Mu) (2), B.1.623 (1), B.1.628 (1), C.37 (Lambda) (3), P.1 (Gamma) (3)). For a 1 × 20 µL qPCR reaction, we used 5 µL of TaqPath 1-Step Multiplex Master Mix, no ROX (Thermo Fisher, Waltham, MA), 0.8 µL each forward and reverse primer from a 10 µM working concentration (final concentration of 400 nM each primer), 0.4 µL of FRET probe from a 10 µM working concentration (final concentration of 200 nM), 8 µL of molecular grade water, and 5 µL of purified sample RNA. RT-qPCR was performed on a QuantStudio 7 instrument. The assay was performed with the following PCR cycling conditions: 25 °C for 2 min, 53 °C for 30 min (reverse transcription, RT), 95 °C for 3 min, and 45 cycles of 95 °C for 10 s and 60 °C for 30 s.

For the Omicron-specific NTD25-7 assay, we used the same reaction and cycling conditions allowing for an eventual transition to a multiplexed assay containing both NTD156-7 and NTD25-7 RT-qPCR assays. As other SARS-CoV-2 variants have been displaced by the emergence of dominant strains, such as Delta and Omicron, we included two Delta lineages (AY.3 and AY.47) which were circulating within our study population at the time, albeit in low numbers (~2% of sequenced samples), and Omicron lineages BA.1, BA.1.1, and BA.2 for the in vitro validation of the Omicron-specific RT-qPCR assay. We followed the same protocol as described above for 56 COVID-19-positive samples previously identified by NGS as AY.3 (*n* = 2), AY.47 (*n*= 1), BA.1 (*n* = 9), BA.1.1 (*n* = 17), and BA.2 (*n* = 27) lineages to assess the performance of the NTD25-7 assay. The TaqPath COVID-19 Combo Kit provides negative, positive, and internal controls to monitor the reliability of the results for the entire batch of specimens from RNA extraction to PCR amplification. According to manufacturer’s instructions, an internal control Ct < 37 is considered positive, and N, ORF1ab, and S Ct >37 is considered negative for SARS-CoV-2. In our study, the Ct value cutoff was <35, and samples lacking amplification (Ct = 0) for at least one of the three targets (except for the S gene target in the case of SGTF) were considered “not evaluable”.

## 3. Results

### 3.1. ARTIC SARS-CoV-2 Sequencing Method Performance Is Directly Related to Sample Ct Values

**Figure 1:**
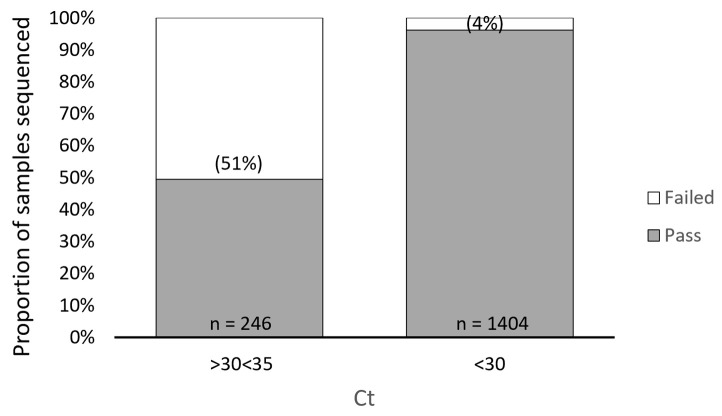
Impact of Ct Value on ARTIC SARS-CoV-2 Sequencing Performance. A retrospective analysis showing 126/246 of sequenced samples with N-gene target Ct values ranging from 30 to 35 (TaqPath COVID-19 Combo Kit RT-qPCR assay) failed, representing a sequencing failure rate of 51%. This failure rate was significantly decreased in samples with Ct values < 30, where only 54/1404 (4%) resulted in sequencing failure.

**Figure 2:**
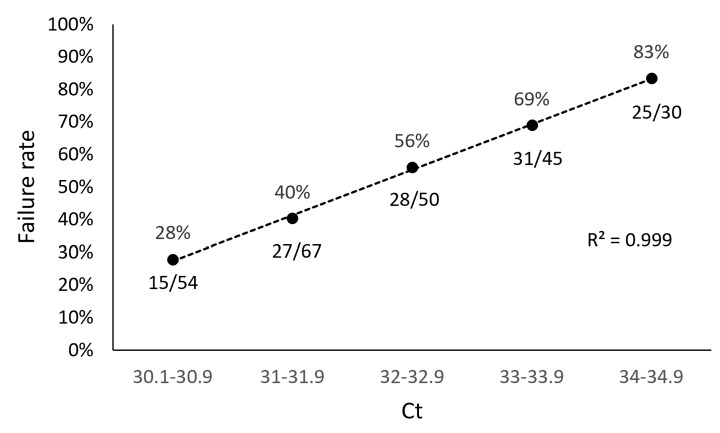
Correlation Between ARTIC SARS-CoV-2 Sequencing Failure Rate and Sample Ct. To determine the impact of Ct value on NGS performance with the ARTIC SARS-CoV-2 sequencing method, 246 sample that failed NGS were grouped into Ct value ranges of 30.1–30.9, 31.0–31.9, 32.0–32.9, 33.0–33.9, and 34.0–34.9. Sequencing failure rate increased in a Ct-dependent manner (correlation R^2^ = 0.999), ranging from 28% (15/54 samples, Ct 30.1–30.9) to 83% (25/30 samples, Ct 34–34.9).

In our study, we found a direct relationship between sample Ct values obtained from the TaqPath assay and sequencing failure rate with the ARTIC method. We performed a retrospective analysis of 1650 samples submitted for NGS and used the TaqPath N-gene target as the reference Ct to determine the relationship between Ct value and sequencing failure rate. The rationale for selecting the N-gene as the reference Ct for this analysis was based on the observation that the Ct values for the ORF1ab and S gene targets (TaqPath) generally trended higher compared to the N gene target, suggesting that the N gene target is more analytically sensitive. Furthermore, the N gene is highly conserved and has a lower mutation rate than the S gene [43,44]. From this retrospective analysis, we found 126/246 of our sequenced samples with N-gene target Ct values ranging from 30 to 35 (TaqPath COVID-19 Combo Kit RT-qPCR assay) failed, representing a sequencing failure rate of 51%. This failure rate was significantly decreased in samples with Ct values < 30, where only 54/1404 (4%) resulted in sequencing failure (Figure 1). To better understand the impact of Ct value on NGS performance with the ARTIC method, we grouped the 246 samples that failed NGS into Ct value ranges of 30.1–30.9, 31.0–31.9, 32.0–32.9, 33.0–33.9, and 34.0–34.9 and found sequencing failure increased in a Ct-dependent manner (correlation R^2^ = 0.999), 28%, 40%, 56%, 69%, and 83%, respectively (Figure 2). 

### 3.2. Targeted RT-qPCR for Detection and Surveillance of VOCs Delta and Omicron

**Figure 3:**
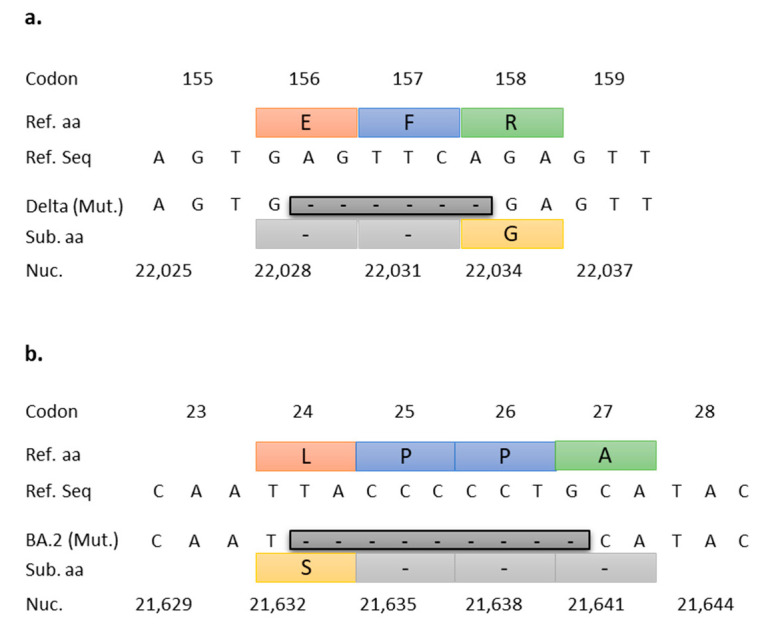
RT-qPCR target deletions for VOCs Delta and Omicron. (**a**) Alignment of Delta E156-F157 deletion with Wuhan reference sequence. This 6 bp deletion (Delta (Mut.)) spans three codons and results in the deletion of amino acids glutamate (E) at position 156 and phenylalanine (F) at position 157, and a non-synonymous substitution at position 158 (arginine (R) for glycine (G)). (**b**) Alignment of Omicron BA.2 lineage P25-P26-A27 deletion (also present in BA.4 and BA.5 lineages) with Wuhan reference sequence. The 9 bp deletion spans four codons resulting in a non-synonymous substitution of leucine (L) with serine (S) at position 24, and deletion of two proline (P) and one alanine (A) residues at positions 25–27. Ref. aa (reference amino acids), Ref. seq (reference sequence), Mut. (Mutation), Sub aa (substitution amino acids), Nuc. (nucleotides).

The NTD156-7 assay was designed to target a highly conserved 6 bp deletion located in the N-terminal-domain (NTD) of the S gene at nucleotide positions 22,029–22,034 corresponding to aa glutamate (E) at position 156 and phenylalanine (F) at position 157 (E156-F157) of the Delta variant, including the AY lineages (Figure 3a). Similarly, the NTD25-7 assay targets a 9 bp deletion in the NTD of the S gene at nucleotide positions 21,633–21,641, which spans four codons resulting in a non-synonymous substitution of leucine (L) with serine (S) at position 24, and deletion of two proline (P) and one alanine (A) residues at positions 25–27 (P25-P26-A27) (Figure 3b).

### 3.3. In Silico Validation Results

Primers and probes for the two targeted RT-qPCR assays (Table 1) were initially validated in silico to ensure a high level of assay performance with minimal requirement for in vitro optimization. The overall rate of mutation for the NTD156-7primers and FRET probe was 1.9%, based on a search of GISAID SARS-CoV-2 sequences deposited over the 6-month search period, 23 February 2021 to 22 August 2021 (1,305,468 sequences) (Table 2). A non-synonymous mutation (glycine to aspartic acid) in the fourth base from the 5-prime end of the forward primer at nucleotide position 21,987 in the NTD of the S gene (associated with the amino acid substitution G142D and seen predominantly in the Delta Plus sub-lineages AY.1 and AY.2) had the highest mutation frequency (1.34%) [44]. This mutation at position 21,987 was not expected to have a significant impact on performance of the forward primer since the remaining 15 bases from the 3-prime end contain very low overall mutation frequencies. To determine the prevalence of this mutation in our study population, we performed an internal review of all Delta variant sequences from our surveillance population and found the G21987 base to be mutated in only 8/395 samples (2%). We tested the 8 samples containing the A21987 genotype (G142D substitution), and the NTD156-7 RT-qPCR assay detected 8/8 samples (CT values < 20). Sequence variability for the 24 -nucleotide FRET probe target revealed very low mutational frequency over the 6-month period analyzed. A nucleotide BLAST (performed 6 September 2021) of the probe, querying Betacoronavirus sequences deposited in the NCBI database, revealed 100% (24/24) concordance with 4997/5000 Delta variant targets, or a variation frequency of 0.06%. In silico validation of the NTD25-7 (Omicron BA.2, BA.4, and BA.5 lineages) primers and FRET probe showed an overall rate of mutation of 3.7%, based on 220,732 GISAID SARS-CoV-2 sequences deposited from 19 December 2021 to 19 March 2022 (Table 3). The forward primer was the oligonucleotide with the highest variability (2.37%); however, the rate of mutation was low within the last five bases at the 3′-end (0.29%). The reverse primer and FRET probe had overall rates of mutation of 1.27% and 0.07%, respectively, with low 3′-end mutation rates of 0.23% (reverse primer) and 0.01% (probe).

The probe for the Delta assay (NTD156-7) was labeled at the 5′-end with the reporter molecule 6-carboxyfluorescein (FAM) and with a double quencher, ZEN (internal), and Iowa Black (3IABkFQ) at the 3′-end. The Omicron-specific assay (NTD25-7) probe is labeled at the 5′-end with the reported Cy5 and with quenchers TAO (internal) and Iowa Black at the 3′-end.

The overall rate of mutation for the NTD156-7 primers and FRET probe was 1.9%, based on a search of GISAID SARS-CoV-2 sequences deposited over the 6-month search period 23 February 2021 to 22 August 2021 (1,305,468 sequences). Reference bases are displayed vertically in a 5′ (top) to 3′ (bottom) orientation.

The overall rate of mutation for the NTD25-7 primers and FRET probe was 3.7%, based on 220,732 SARS-CoV-2 sequences deposited in GISAID from 19 December 2021 to 19 March 2022. Reference bases are displayed vertically in a 5′ (top) to 3′ (bottom) orientation.

### 3.4. In Vitro Validation Results

The performance of the NTD156-7assay was tested on 50 samples identified previously by NGS as Delta variants as well as an assortment of samples identified previously by NGS as non-Delta variants (see methods). The NTD156-7 RT-qPCR assay correctly identified 24/24 Delta variant samples with Ct values ranging from 20.19 to 38.6 (mean Ct = 28.43) representing a positive percent agreement (PPA) of 100%. The assay showed no amplification for 26/26 non-Delta variant samples of different lineages, representing a negative percent agreement (NPA) of 100% (Table 4). The NTD25-7 RT-qPCR assay performed equally well in vitro. Of the 53 BA.2 lineages (BA.2 (51), BA.2.3 (2)) previously identified by NGS, 53 showed amplification by our targeted RT-qPCR assay (mean CT = 27.2 and 20.1, respectively) (PPA = 100%), and 29/29 non-BA.2 lineage samples by NGS showed no amplification (NPA = 100%) (Table 5). Beyond the initial in vitro validation of the two targeted RT-qPCR assays, we evaluated assay performance on an ongoing basis. Both assays continued to demonstrate 100% PPA/NPA, as confirmed by randomly selected samples for NGS characterization.

Of the diverse variants previously identified by NGS, 50 samples were selected for the initial validation. Delta variant samples (*n* = 24) include B.1.617.2 (9), AY.2 (1), AY.3 (3), AY.3.1 (2), AY.4 (1), AY.5 (1), AY.12 (1), AY.24 (1), and AY.25 (5). Non-Delta samples (*n* = 26) comprise B.1 (1), B.1.1.7 (Alpha) (6), B.1.427 (2), B.1.429 (2), B.1.526 (3), B.1.621 (Mu) (2), B.1.621.1 (Mu) (2), B.1.623 (1), B.1.628 (1), C.37 (Lambda) (3), and P.1 (Gamma) (3). The NTD156-7 RT-qPCR assay correctly identified 24/24 Delta variant samples (positive percent agreement (PPA) = 100%) and showed np amplification (na) for 26/26 non-Delta variant samples of different lineages (negative percent agreement (NPA) = 100%). Observed CT values for Delta variant samples ranged from 20.19 to 38.6.

The BA.2-specific RT-qPCR assay correctly identified 53/53 BA.2 (*n* = 51) and BA.2.3 (*n* = 2) variants (PPA = 100%) and showed no amplification (na) for 29/29 (NPA = 100%) non-BA.2 lineages (BA.1 (9), BA.1.1 (17), AY.3 (2), and AY.47 (1)).

### 3.5. SARS-CoV-2 VOC Surveillance Workflow

**Figure 4:**
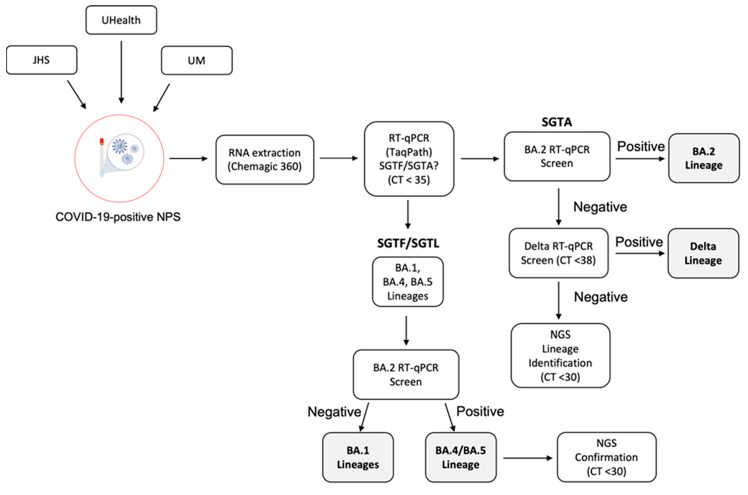
SARS-CoV-2 VOC Surveillance Workflow. In the workflow depicted, nasopharyngeal swab (NPS) samples were obtained from Jackson Health System (JHS), University of Miami Health System (UHealth), and University of Miami (UM) for variant identification. The first RT-qPCR assay (TaqPath) serves to identify SGTF/SGTL associated with the H69-V70 deletion present in the Omicron BA.1, BA.4, and BA.5 lineages. For this assay, our N gene target Ct cutoff was 35, with similar amplification (<2 Ct difference) for the ORF1ab and S gene targets in samples showing S gene target amplification (SGTA) and the same rule applied for the ORF1ab gene target in SGTF/SGTL samples. Next, samples (SGTF/SGTL and SGTA) were screened with the NTD25-7 RT-qPCR assay, for this and the Delta assay we were able to increase the Ct cutoff to 38 as we found reliable and reproducible results within this Ct range. Samples positive with the NTD25-7 assay, which also exhibit SGTF/SGTL, were presumed BA.4 or BA.5 lineages and were subsequently sequenced for lineage confirmation. SGTF/SGTL samples negative with the NTD25-7 assay are presumed BA.1 lineage. SGTA samples positive with the NTD25-7 assay were presumed BA.2 lineage. BA.2-negative, SGTA samples were subsequently screened for the Delta variant by targeted RT-qPCR (NTD156-7). If negative for Delta, these samples were sequenced for lineage identification.

**Figure 5:**
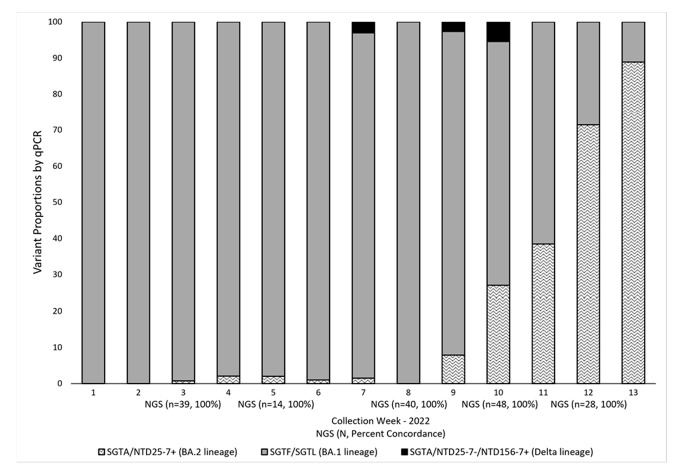
SARS-COV-2 Variant Proportions by RT-qPCR and NGS. Variant proportions were determined by RT-qPCR and NGS in residual COVID-19-positive samples collected from week 1 (1/3–1/9) to week 13 (3/23–4/1), 2022. Variant surveillance by RT-qPCR (*n* = 976) was performed weekly using the TaqPath assay in combination with internally designed and validated targeted RT-qPCR assays (NTD156-7 and NTD25-7). Subsets of these samples (*n* = 169) (Ct < 30) were sequenced on weeks 3 (*n* = 39), 5 (*n* = 14), 8 (*n* = 40), 10 (*n* = 48), and 12 (*n* = 28). Periodic confirmation by NGS of results obtained by RT-qPCR screenings provided confidence in the ongoing variant surveillance by targeted RT-qPCR methods. For the five sequencing timepoints illustrated, concordance between NGS and RT-qPCR results was 100%. SGTA (S-gene target amplification detected with the TaqPath assay); NTD25-7 (RT-qPCR assay targeting the N-terminal-domain deletion corresponding to amino acids 25–27 of the spike (S) protein in Omicron BA.2 lineages); SGTF/SGTL (S-gene target failure/S-gene target late amplification (TaqPath) corresponding to NTD deletion 69–70 of the S protein in Omicron BA.1 lineages); NTD156-7 (RT-qPCR assay targeting the NTD deletion corresponding to amino acids 156–157 of the S protein in the Delta lineage).

The current workflow for SARS-CoV-2 variant identification and surveillance (Figure 4) can effectively detect Omicron BA.1, BA.2, and BA.4/BA.5 lineages and the Delta variant by RT-qPCR methods. The initial RT-qPCR assay (TaqPath) serves as a quality assurance step to confirm positivity and assess sample integrity. In addition, this step allows us to identify samples with N gene target Ct values < 30 as a criterion for NGS. In the process of variant identification, the first RT-qPCR assay (TaqPath) serves to identify SGTF/SGTL samples associated with the H69-V70 deletion present in the Omicron BA.1, BA.4, and BA.5 lineages. For this assay, our N gene target Ct cutoff is 35, with similar amplification (<2 Ct difference) for the ORF1ab and S gene targets in samples showing S gene target amplification (SGTA) and the same rule applies for the ORF1ab gene target in SGTF/SGTL samples. Next, samples (SGTF/SGTL and SGTA) were screened with the NTD25-7 RT-qPCR assay. The Ct value cutoff for this and the NTD156-7 assay was <38, as we observed variant confirmation by NGS within this Ct range. Samples positive with the NTD25-7 assay that also exhibit SGTF/SGTL are presumed BA.4 or BA.5 lineages, with lineage confirmation established by NGS. The SGTF/SGTL samples found to be negative with the NTD25-7 assay are presumed BA.1 lineage. Samples exhibiting SGTA and positive with the NTD25-7 assay were presumed BA.2 lineage. Non-BA.2 SGTA samples were subsequently screened for the Delta variant by targeted RT-qPCR (NTD156-7). If negative, these samples were sequenced for lineage identification. Figure 5 provides a snapshot of how we integrated NGS into variant prevalence monitoring by PCR for the first 13 weeks of 2022, when Omicron BA.1 was outcompeted and eventually replaced by the BA.2 lineage. Over this period, we performed 976 RT-qPCR reactions, typically with one or two runs per week and selected a subset of samples (*n* = 169) for periodic NGS confirmation. The results from NGS analysis were in complete concordance with our RT-qPCR conclusions and provided ongoing validation of our targeted RT-qPCR methods. For the five sequencing timepoints illustrated in Figure 5, concordance between NGS and RT-qPCR results was 100%.

## 4. Discussion

The targeted RT-qPCR assays presented here for detection and surveillance of VOCs Delta and Omicron in combination with the TaqPath assay for identification of Omicron lineages harboring the H69-V70 deletion, resulting in the characteristic SGTF/SGTL signature, provide a simple and cost-effective alternative for near real-time variant identification, which has important clinical implications. While NGS remains the gold standard for genomic characterization of SARS-CoV-2 variants, the widely used ARTIC SARS-CoV-2 sequencing method has inherent limitations including high failure rates for samples with Ct values > 30, which can dampen variant surveillance efforts by excluding samples with lower viral loads from genomic characterization. Another limitation associated with this sequencing approach is primer/variant overlap in lineages with heavily mutated S genes, as is the case with the Omicron lineages. Primer/variant overlap results in amplification failure and subsequent overall decrease in NGS coverage. In addition to these limitations, NGS is cost- and labor-intensive, not readily available in many regions, and is associated with slower workflows compared to qPCR-based methods. It is important to note that the PCR methods proposed here do not exclude NGS as we recognize its tremendous value in providing ongoing confirmation that our qPCR assays are accurately reflecting local variant prevalence. Given the dynamic nature of SARS-CoV-2 and its ongoing genetic evolution, it is important to perform periodic method quality checks by randomly selecting samples screened by qPCR to be confirmed by sequencing. Sequencing methods are able to detect changes in areas that are distant and distinct genomic locations from the primer/probe binding sites. This underscores the importance of periodic random sequencing of samples. This combined approach significantly reduces the cost of variant surveillance while providing ongoing validation of targeted qPCR methods.

From January 2021 to February 2022, we performed RT-qPCR-based variant screening of over 5000 COVID-19 positive samples, of which 1668 were genetically characterized by NGS. This two-pronged approach allowed for cost-effective utilization of sequencing resources and continuous tracking of the prevalence and dynamics of locally circulating SARS-COV-2 variants. Ultimately, our efforts helped inform local public and private health systems, and University of Miami leaders’ decisions in their efforts to ameliorate the spread of COVID-19 in the Miami-Dade County community and allocate resources including staffing in preparation for hospitalization surges. Variant monitoring also allowed our antimicrobial stewardship and infection prevention programs to target appropriate isolation policies and therapeutics. As some monoclonals became ineffective, we were able to pivot our hospital and outpatient formularies to provide appropriate therapies based on risk factors and therapeutic effectiveness. In addition to monitoring variant prevalence and dynamics by qPCR and NGS, we also followed national and Miami-Dade County variant prevalence data available from the CDC COVID Data Tracker. The VOC prevalence observed in our cohort mirrored closely the CDC Variant Proportions reports. Our combined qPCR and NGS approach provided information that was directly relevant to our surveillance population and was available in near real time. Epidemiologically, continuous monitoring of the prevalence and dynamics of circulating variants can better inform local health leaders’ decisions to adopt preventive measures to control community transmission of COVID-19 infections.

Additionally, by incorporating tracking of variant dynamics by RT-qPCR-based methods, near real-time changes in variant prevalence can be determined, with subsequent confirmation by NGS. The continued genetic evolution of SARS-CoV-2 and the rapid emergence and rise to dominance of new VOCs with different mutational landscapes underscores the value of an equally dynamic surveillance approach. For this reason, revision of variant tracking workflows is necessary to account for these genetic variations. New targeted qPCR assays may need to be developed and implemented as part of the workflow as novel variant-defining mutations emerge. Surveillance accuracy will be enhanced by incorporating several targeted qPCR assays in communities where multiple dominant variants circulate. Despite these limitations, in addition to providing a rapid, cost-effective alternative for tracking the prevalence of SARS-CoV-2 variants, RT-qPCR-based surveillance strategies permit better utilization of NGS resources by avoiding sequencing redundancy (and associated investment of resources) in an environment where a dominant variant prevails in the population. In addition, PCR approaches provide a feasible option for variant surveillance in communities where NGS is not readily available. Importantly, rapid near-real-time emergence of known variants not yet circulating can be accomplished more easily with PCR approaches in conjunction with sequencing support. Confidence in calling variant progression by PCR is made possible by regular NGS analysis of subsets of samples. Future studies should address the balance between qPCR surveillance methods and parallel NGS analysis for optimal accuracy of variant calling. Therefore, RT-qPCR serves as complementary methods to allow active, accurate identification and surveillance of changes in variant prevalence over time that can impact public health and improve direct patient care. The ability to detect changes in VOC prevalence in our surveillance population reinforced messaging to the community regarding the importance of vaccination uptake and vigilance. VOC prevalence (especially BA.2) informed strategic decisions regarding anti-SARS-CoV-2 monoclonal antibody therapies (e.g., sotrovimab) to include in hospital formularies. By having active VOC prevalence available, health system leaders were able to make critical decisions with greater confidence and in a timelier manner.

## Figures and Tables

**Table 1:** Primers and probes for targeted RT-qPCR assays.

	Primer/Probe	Sequence 5′-3′
Delta (NTD156-7)	Forward Primer	TGGGTGTTTATTACCACAA
	Reverse Primer	GGCTGAGAGACATATTCAAA
	Probe	FAM-ATGGAAAGT/ZEN/GGAGTTTATTCTAGT-3IABkFQ
Omicron BA.2, BA.4, BA.5 (NTD25-7)	Forward Primer	TTTATTGCCACTAGTCTCTAGTCAG
	Reverse Primer	GGTAATAAACACCACGTGTGAAAG
	Probe	Cy5-AGAACTCAA/TAO/TCATACACTAATT-3IAbRQSp

**Table 2:** In silico Validation of NTD156-7 (Delta) Primers and Probe.

	NTD156-7 Forward Primer	Mutated Sequences		NTD156-7 Reverse Primer	Mutated Sequences		NTD156-7 Probe	Mutated Sequences	
	Ref Base	#	%	Ref Base	#	%	Ref Base	#	%
5′	T	48	0.004%	G	40	0.003%	A	196	0.015%
	G	3	0.000%	G	722	0.055%	T	187	0.014%
	G	4	0.000%	C	638	0.049%	G	1011	0.077%
	G	17,428	1.335%	T	68	0.005%	G	16	0.001%
	T	0	0.000%	G	346	0.027%	A	35	0.003%
	G	2	0.000%	A	512	0.039%	A	10	0.001%
	T	28	0.002%	G	77	0.006%	A	31	0.002%
	T	30	0.002%	A	455	0.035%	G	126	0.010%
	T	15	0.001%	G	45	0.003%	T	251	0.019%
	A	152	0.012%	A	19	0.001%	G	274	0.021%
	T	76	0.006%	C	142	0.011%	G	57	0.004%
	T	15	0.001%	A	142	0.011%	A	60	0.005%
	A	0	0.000%	T	4	0.000%	G	22	0.002%
	C	3	0.000%	A	156	0.012%	T	26	0.002%
	C	3	0.000%	T	36	0.003%	T	35	0.003%
	A	2	0.000%	T	7	0.001%	T	34	0.003%
	C	3	0.000%	C	12	0.001%	A	3	0.000%
	A	2	0.000%	A	10	0.001%	T	60	0.005%
	A	29	0.002%	A	181	0.014%	T	12	0.001%
				A	447	0.034%	C	54	0.004%
							T	18	0.001%
							A	20	0.002%
							G	149	0.011%
3′							T	29	0.002%
NTD156-7 Oligos		Sequence 5′-3′			% Sequences with any mutation		% Sequences with mutation in last 5 bases		
Forward Primer		TGGGTGTTTATTACCACAA			1.367		0.003		
Reverse Primer		GGCTGAGAGACATATTCAAA			0.311		0.050		
Probe		ATGGAAAGTGGAGTTTATTCTAGT			0.210		0.020		

**Table 3:** In silico Validation of NTD25-7 (Omicron) Primers and Probe.

	NTD25-7 Forward Primer	Mutated Sequences		NTD25-7 Reverse Primer	Mutated Sequences		NTD25-7 Probe	Mutated Sequences	
	Ref Base	#	%	Ref Base	#	%	Ref Base	#	%
5′	T	18	0.008%	G	24	0.011%	A	0	0.000%
	T	12	0.005%	G	385	0.174%	G	15	0.007%
	T	6	0.003%	T	19	0.009%	A	0	0.000%
	A	32	0.014%	A	12	0.005%	A	0	0.000%
	T	27	0.012%	A	81	0.037%	C	3	0.001%
	T	4	0.002%	T	284	0.129%	T	34	0.015%
	G	69	0.031%	A	281	0.127%	C	1	0.000%
	C	18	0.008%	A	9	0.004%	A	0	0.000%
	C	31	0.014%	A	6	0.003%	A	1	0.000%
	A	28	0.013%	C	926	0.420%	T	45	0.020%
	C	48	0.022%	A	8	0.004%	C	2	0.001%
	T	3	0.001%	C	8	0.004%	A	1	0.000%
	A	8	0.004%	C	10	0.005%	T	3	0.001%
	G	31	0.014%	A	9	0.004%	A	4	0.002%
	T	18	0.008%	C	14	0.006%	C	14	0.006%
	C	3672	1.664%	G	5	0.002%	A	0	0.000%
	T	17	0.008%	T	15	0.007%	C	8	0.004%
	C	480	0.217%	G	134	0.061%	T	12	0.005%
	T	38	0.017%	T	66	0.030%	A	0	0.000%
	A	31	0.014%	G	337	0.153%	A	1	0.000%
	G	242	0.110%	A	135	0.061%	T	12	0.005%
	T	33	0.015%	A	9	0.004%	T	0	0.000%
	C	30	0.014%	A	13	0.006%			
	A	52	0.024%	G	2	0.001%			
3′	G	285	0.129%						
NTD25-7 Oligos		Sequence 5′-3′			% Sequences with any mutation		% Sequences with mutation in last 5 bases		
Forward Primer		TTTATTGCCACTAGTCTCTAGTCAG			2.371		0.291		
Reverse Primer		GGTAATAAACACCACGTGTGAAAG			1.265		0.225		
Probe		AGAACTCAATCATACACTAATT			0.071		0.011		

**Table 4:** In vitro Validation of NTD156-7 Assay.

Variant (NGS)	*n*	NTD156-7 Result		Mean Ct	Percent Agreement
		Positive	Negative		
B.1.617.2 (Delta)	9	9	0	28.60	100%
AY.2 (Delta)	1	1	0	25.20	100%
AY.3 (Delta)	3	3	0	28.90	100%
AY.3.1 (Delta)	2	2	0	31.05	100%
AY.4 (Delta)	1	1	0	26.94	100%
AY.5 (Delta)	1	1	0	37.73	100%
AY.12 (Delta)	1	1	0	26.77	100%
AY.24 (Delta)	1	1	0	20.19	100%
AY.25 (Delta)	5	5	0	27.86	100%
B.1	1	0	1	na	100%
B.1.1.7 (Alpha)	6	0	6	na	100%
B.1.427 (Epsilon)	2	0	2	na	100%
B.1.429 (Epsilon)	2	0	2	na	100%
B.1.526 (Iota)	3	0	3	na	100%
B.1.621 (Mu)	2	0	2	na	100%
B.1.621.1 (Mu)	2	0	2	na	100%
B.1.623	1	0	1	na	100%
B.1.628	1	0	1	na	100%
C.37 (Lambda)	3	0	3	na	100%
P.1 (Gamma)	3	0	3	na	100%
Total	50	24	26		

**Table 5:** In vitro Validation of the NTD25-7 Assay.

Variant (NGS)	*n*	NTD25-7 Results		Mean Ct	Percent Agreement
		Positive	Negative		
BA.2 (Omicron)	51	51	0	27.2	100%
BA.2.3	2	2	0	20.1	100%
BA.1 (Omicron)	9	0	9	na	100%
BA.1.1 (Omicron)	17	0	17	na	100%
AY.3 (Delta)	2	0	2	na	100%
AY.47 (Delta)	1	0	1	na	100%
Total	82	53	29		
